# Soluble Fluorinated Cardo Copolyimide as an Effective Additive to Photopolymerizable Compositions Based on Di(meth)acrylates: Application for Highly Thermostable Primary Protective Coating of Silica Optical Fiber

**DOI:** 10.3390/ijms25105494

**Published:** 2024-05-17

**Authors:** Dmitriy A. Sapozhnikov, Olga A. Melnik, Alexander V. Chuchalov, Roman S. Kovylin, Sergey A. Chesnokov, Dmitriy A. Khanin, Galina G. Nikiforova, Alexey F. Kosolapov, Sergey L. Semjonov, Yakov S. Vygodskii

**Affiliations:** 1 A. N. Nesmeyanov Institute of Organoelement Compounds, Russian Academy of Sciences, Vavilov Str. 28, Moscow 119334, Russia; omel@ineos.ac.ru (O.A.M.); stormrage969@gmail.com (A.V.C.); d.a.khanin@gmail.com (D.A.K.); ggn@ineos.ac.ru (G.G.N.); yasvyg@ineos.ac.ru (Y.S.V.); 2 G. A. Razuvaev Institute of Organometallic Chemistry, Russian Academy of Sciences, Tropinin Str. 49, Nizhniy Novgorod 603950, Russia; mulnir@yandex.ru (R.S.K.); sch@iomc.ras.ru (S.A.C.); 3 Department of Macromolecular Compounds and Colloid Chemistry, National Research Lobachevsky State University of Nizhniy Novgorod, Gagarin Ave. 23, Nizhniy Novgorod 603022, Russia; 4 Dianov Fiber Optics Research Center, Prokhorov General Physics Institute of the Russian Academy of Sciences, Vavilov Str. 38, Moscow 119333, Russia; kaf@fo.gpi.ru (A.F.K.); sls@fo.gpi.ru (S.L.S.)

**Keywords:** UV photopolymerization, di(meth)acrylates, polyimide, protective coating, silica optical fiber, high thermal stability

## Abstract

The development of photocurable compositions is in high demand for the manufacture of functional materials for electronics, optics, medicine, energy, etc. The properties of the final photo-cured material are primarily determined by the initial mixture, which needs to be tuned for each application. In this study we propose to use simple systems based on di(meth)acrylate, polyimide and photoinitiator for the preparation of new photo-curable compositions. It was established that a fluorinated cardo copolyimide (FCPI) based on 2,2-*bis*-(3,4-dicarboxydiphenyl)hexafluoropropane dianhydride, 9,9-*bis*-(4-aminophenyl)fluorene and 2,2-*bis*-(4-aminophenyl)hexafluoropropane (1.00:0.75:0.25 mol) has excellent solubility in di(met)acrylates. This made it possible to prepare solutions of FCPI in such monomers, to study the effect of FCPI on the kinetics of their photopolymerization in situ and the properties of the resulting polymers. According to the obtained data, the solutions of FCPI (23 wt.%) in 1,4-butanediol diacrylate (BDDA) and FCPI (15 wt.%) in tetraethylene glycol diacrylate were tested for the formation of the primary protective coatings of the silica optical fibers. It was found that the new coating of poly(BDDA–FCPI_23%_) can withstand prolonged annealing at 200 °C (72 h), which is comparable or superior to the known most thermally stable photo-curable coatings. The proposed approach can be applied to obtain other functional materials.

## 1. Introduction

Photopolymerization is a widely used method for converting a liquid composition into a solid polymer when exposed to UV, visible or IR light [1,2,3]. The main advantage of radical photopolymerization is the ability to cure compositions that do not contain inert solvents in seconds under normal conditions. This process is faster and more energy efficient than thermal radical polymerization. The abundance of possible photopolymerizable compositions, as well as the obvious advantages of their transformation and the rapidly developing technologies of 3D-printing, make them attractive for the formation of a variety of functional materials [4,5,6,7].

Photo-curable compositions have been extensively used to produce materials for various applications including in regenerative medicine [8,9], in particular, multifunctional hydrogel dressings [9], biomimetic magnetic structures [10], composites with antimicrobial properties provided by the inclusion of quaternary ammonium salts [11], and materials against Staphylococcus aureus [12]. Recent advances have made it possible to design and print dentures, crowns and mouthguards [13,14,15]. Achievements in this field allow the use of modern 3D-printing methods for the production of tissue-engineered constructs [16,17,18] and drug delivery systems [18,19,20,21]. Photo-curable coatings are used, among other things, in the manufacture of various electronic and medical devices, optical fibers, etc. [22,23,24]. Most of the curable compositions contain multifunctional (meth)acrylates—di- and tri(meth)acrylates. Such compounds, unlike non-functional unsaturated monomers, do not require thermal post-curing [25], which is a favorable feature. Polyurethane/polysiloxane/polyether acrylates [22,26] or expensive fluoroacrylates [22] are used to achieve high thermal stability of photo-cured silica optical fiber coatings. The use of such initial systems has its own peculiarities and limitations, including synthetic ones.

Polyimides (PIs) are an important class of condensation polymers characterized by high thermal stability and excellent mechanical properties [27,28,29,30,31,32,33]. There are approaches to obtain biocompatible materials by the modification of implantable polyimide films [34] and polyimide covalent organic frameworks for drug delivery applications [35]. Photosensitive compositions based on aromatic heterochain polymers and reactive solvents can be used to form mechanically durable three-dimensional objects by laser stereolithography [36,37,38,39,40,41], digital light processing (DLP) [42,43,44,45,46,47] and liquid crystal display (LCD) [48,49,50] 3D printing.

Previously, we performed investigations of the free radical thermal homo- and copolymerization of (meth)acrylates, styrene and N-vinyl-2-pyrrolidone containing dissolved polyheteroarylenes including PIs [51,52,53,54,55]. It was found that the obtained polymer systems differ from the corresponding homopolymers in terms of molecular weight characteristics and thermal and mechanical properties. The study of the kinetics of free radical thermal and photopolymerization (meth)acrylates in the presence of polyimide, polyarylate and their model compounds by differential scanning photocalorimetry, FTIR, ESR spectroscopy, etc. [51,53,54,55,56] allowed us to identify the mechanism of copolymer formation as the chain transfer reaction to functional groups of polyheteroarylenes, in particular to imide cycles [54,56]. The introduction of polyheteroarylenes into the monomer(s) and subsequent polymerization in situ contributed to an increase in the thermal and mechanical properties of the copolymers formed compared to unmodified carbon-chain homopolymers [51,52,53,54,55,56], opening up wide possibilities for obtaining various functional materials.

In this work we demonstrate a new route for the development of functional photopolymerizable compositions (PPCs) applicable to the formation of highly thermostable coatings of silica optical fibers. A simple three-component photopolymerizable composition is formed by dissolving highly fluorinated cardo copolyimide in di(meth)acrylate and adding a photoinitiator. The use of only bifunctional monomers has advantages over monofunctional ones, which are indicated above. The kinetics of the photopolymerization of di(meth)acrylates containing dissolved polyimide and the properties of photo-cured compositions have been studied and PPCs suitable for the formation of a primary protective coating of a silica optical fiber have been selected. It is shown that the coating based on 1,4-butanediol diacrylate and 23 wt.% copolyimide can withstand prolonged annealing at 200 °C. The developed compositions can be considered for the production of special optical fibers including medical ones.

## 2. Results and Discussion

A fluorinated cardo copolyimide (FCPI), which is highly soluble in monofunctional (meth)acrylates, was synthesized by a one-step high-temperature polycondensation of 2,2-bis-(3,4-dicarboxydiphenyl)hexafluoropropane dianhydride, 9,9-bis-(4-aminophenyl)fluorene and 2,2-bis-(4-aminophenyl)hexafluoropropane (1.00:0.75:0.25 mol) in *m*-cresol solution [57] to obtain photo-curable varnishes based on di(meth)acrylates and polyimide. In order to achieve the maximum polymerization rate and obtain a mixture of the required viscosity with maximum filling, FCPI with relatively low molecular weight properties (η_inh_ = 0.36 dLg^−1^, M_n_ = 36 kDa, M_w_ = 56 kDa) was synthesized. Previously, using the example of methyl methacrylate polymerization in the presence of FCPI characterized by different molecular weights, it was shown that the maximum values of the polymerization rates of this monomer are obtained at a lower molecular weight of the PI [55]. Furthermore, the introduction of a low molecular weight PI also leads to an increase in the performance characteristics of the resulting copolymers as well [56].

### 2.1. Photopolymerizing Compositions Based on Di(meth)acrylates and FCPI

The solubility of FCPI in various di(meth)acrylates and the commercial acrylate varnish DeSolite 3471-3-14 (for comparison) was investigated. It was found that the synthesized FCPI is insoluble in DeSolite 3471-3-14, but it is well soluble in 1,4-butanediol diacrylate (BDDA), 1,4-butanediol dimethacrylate (BDDMA), ethylene glycol dimethacrylate (EGDM), tetraethylene glycol diacrylate (TTEGDA), triethylene glycol dimethacrylate (TIEGDMA), and tetraethylene glycol dimethacrylate (TTEGDMA). This made it possible to study the effect of FCPI dissolved in the monomer on its photopolymerization kinetics and on the properties of the formulated polymers. In this way, the properties of the initial PPC, including the desired viscosity of the solution and its reactivity, can be clearly adjusted. In addition, the proposed approach to formulate photo-curable coatings using PIs differs favorably from analogues based on the additional functionalization of the PI macromolecules by methacrylic groups [58] or the use of PI-solutions with an inactive solvent that should be effectively removed [57,59].

#### 2.1.1. Kinetics of Photopolymerization

**Figure 1:**
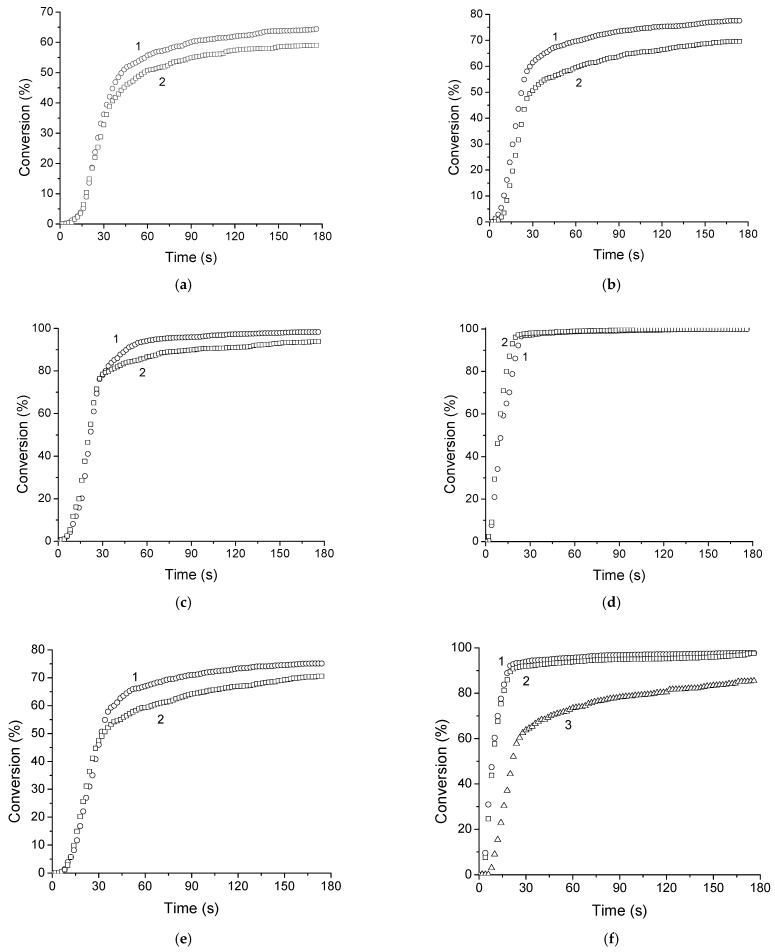
Kinetic curves of photopolymerization: (**a**) EGDMA (1) and 15 wt.% FCPI solution in EGDMA (2); (**b**) TIEGDMA (1) and 15 wt.% FCPI solution in TIEGDMA (2); (**c**) TTEGDMA (1) and 15 wt.% FCPI solution in TTEGDMA (2); (**d**) TTEGDA (1) and 15 wt.% FCPI solution in TTEGDA (2); (**e**) BDDMA (1) and 15 wt.% FCPI solution in BDDMA (2); and (**f**) BDDA (1) and 15 (2), and 23 wt.% FCPI solution in BDDA (3). All PPs contain 5 wt.% DMPA photoinitiator per monomer.

The photopolymerization of all di(meth)acrylates and di(meth)acrylate–FCPI compositions was evaluated relative to each other. It is important to note that this set of monomers makes it possible to study the changes in photopolymerization parameters as the number of ethoxy-groups in dimethacrylate increases (EGDMA-TIEGDMA-TTEGDMA) and also to compare pairs of dimethacrylate-diacrylate monomers with the same spacer between reactive groups (TTEGDMA-TTEGDA and BDDMA-BDDA). 2,2-Dimethoxy-2-phenylacetophenone (DMPA) was used as a photoinitiator. The kinetic parameters of the photopolymerization and the kinetic curves are shown in Table 1 and Figure 1.

It was found that for a series of dimethacrylates, as the number of ethoxy groups in the dimethacrylate grows from one (EGDMA) to three (TIEGDMA) and four (TTEGDMA), the maximum polymerization rate increases by 1.4 and 2.1 times and the maximum conversion increases from 64 to 77 and 98%, respectively. The addition of 15 wt.% FCPI to the methacrylates results in a slight decrease in the limiting conversion (by 4–7%) and a more significant change in the maximum polymerization rate (Table 1). The reduction in the maximum polymerization rate of EGDMA–FCPI_15%_ compared to EGDMA is the most noticeable and is 1.3 times. For TIEGDMA–FCPI_15%_ and TTEGDMA–FCPI_15%_, which contain monomers with longer spacers, this decrease is less pronounced (1.15 times).

It is well known that acrylates are more active in free radical polymerization reactions than corresponding methacrylates [60,61], although there are exceptions [62]. For the studied monomeric dimethacrylate-diacrylate pairs with the same spacer between the reactive (meth)acrylate groups, acrylates polymerize faster than the corresponding methacrylates. Thus, for a pair of TTEGDMA-TTEGDA, the maximum polymerization rate of the diacrylate is 1.38 times higher, and for the BDDMA-BDDA pair it is 3.21 times (Table 1). The limiting conversions for the acrylates studied are higher than for the corresponding dimethacrylates, reaching 100%. The dissolution of FCPI in the monomers has different effects on the polymerization of the corresponding methacrylates and acrylates. The introduction of 15 wt.% FCPI into TTEGDMA reduces its maximum photopolymerization rate by 1.13 times, and an increase in the maximum rate by 1.24 times is observed for the corresponding TTEGDA. When 15 wt.% FCPI is added to di(meth)acrylates with a 1,4-butanediol spacer, the photopolymerization rate for both BDDMA and BDDA increases by 1.13 and 1.02 times, respectively, and the limiting conversion is reduced by no more than 5%. As will be noted below, the solution of 15 wt.% FCPI in PPC based on BDDA does not have the viscosity required to produce a high-quality fiber optic coating. Increasing the concentration of FCPI up to 23 wt.% reduces the maximum photopolymerization rate by 2.11 times and the limiting conversion by 12% compared to pure BDDA. This is most likely due to the limitations imposed by the high-viscosity of the initial solution.

Previously, the effects of different molecular weights of dissolved FCPI and its concentration on the kinetics of the polymerization of methyl methacrylate (MMA) and ethyl acrylate were studied [55,56]. The dissolution of 4 wt.% FCPI with η_inh_ = 0.52 dL/g in MMA led to an overall increase in the polymerization rate of MMA and an earlier gel effect. However, the same amount of PI with a η_inh_ equal to 1.34 dL/g caused a significant slowdown in the reaction even compared to pure MMA. A decrease in the polymerization rate of MMA at all stages was a consequence of a significant increase in the viscosity of the initial solution, which reduced the initiation efficiency by enhancing the cage effect, slowed down the rate of radical diffusion and, respectively, the chain growth and termination reactions [55]. An increase in the concentration of low molecular weight FCPI from 5 to 42 wt.% minimized the polymerization rate of the monomers as well [56].

#### 2.1.2. Properties of Photo-Cured Compositions

Based on previous results [51,52,56], it was suggested that the introduction of FCPI into di(meth)acrylates will improve the thermal stability and strength properties of corresponding polymer compositions, which is important for the formation of protective coatings of silica optical fibers. To investigate these properties, films were prepared from unmodified monomers and their blends with FCPI (see the details in the experimental section). For comparison, a polymer film of the commercial acrylic varnish DeSolite 3471-3-14 was prepared and tested. The values of the tensile strength (σ), modulus of elasticity (E), elongation at break (ε) and onset temperature of thermal decomposition (T_onset_) for the polymers studied are given in Table 2.

**Figure 2:**
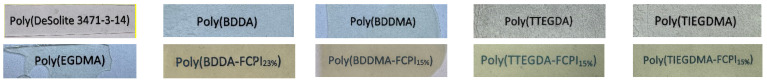
Optical properties of cured polymers.

As can be seen from Figure 2, the addition of FCPI leads to a slight coloring and clouding of the samples. Only poly(EGDMA–FCPI_15%_) and poly(BDDMA–FCPI_15%_) form brittle films (Table 2).

From the mechanical test results presented in Table 2, it is obvious that the poly(DeSolite 3471-3-14) film is characterized by the highest elongation at break (81.0%) and the lowest modulus of elasticity (0.1 GPa) of all of the samples tested. At the same time, the tensile strength of the poly(DeSolite 3471-3-14) is close to that of all di(meth)acrylate-based homopolymers without FCPI.

Comparing poly(EGDMA) and poly(TIEGDMA), the tensile strength of the polymer increases by 1.2 times, and the modulus of elasticity decreases by 25% as the number of ethoxy groups changes from one to three. As previously observed for other PI-containing polymer systems [51,54], the dissolution of 15 wt.% FCPI in TIEGDMA results in a polymer with significantly increased tensile strength (1.5 times) and elongation at break (1.7 times) compared to unmodified poly(TIEGDMA) (Table 2). Among the polymers studied, the mechanical properties of poly(TTEGDA) are most similar to those of poly(DeSolite 3471-3-14), which has a tensile strength of 19.4 MPa, a Young’s modulus of 0.5 GPa and a maximum elongation of 17.5%. It is noteworthy that the addition of 15 wt.% FCPI to TTEGDA resulted in a 27% increase in the tensile strength of poly(TTEGDA–FCPI_15%_) and a 2-fold increase in the elastic modulus, but this was accompanied by a 3.7-fold decrease in the elongation at break. This means that poly(TTEGDA–FCPI_15%_) is more durable and less elastic than poly(TTEGDA), which is in agreement with previous results [51,54]. The most interesting results were obtained for BDDA-based polymers. The introduction of 23 wt.% FCPI into BDDA allowed an enhancement of the tensile strength of the corresponding polymer from 20.2 to 44.0 MPa (2.2 times), the elongation at break from 1.6 to 3.6% (2.2 times) and the modulus of elasticity from 1.5 to 1.9 GPa (1.3 times) (Table 2). Such significant changes in mechanical properties can be caused not only by the presence of rigid-chain FCPI macromolecules in the regions between the crosslinking of the polydi(met)acrylate, but also by the covalent bonding of FCPI macromolecules to a three-dimensional network. This may occur as a result of chain transfer reactions during the free radical polymerization of di(met)acrylates. Such an effect has been previously noted when using methyl methacrylate, ethylacrylate, styrene, N-vinyl-2-pyrrolidone, etc. as a monomer [51,52,53,54,55]. The formation of covalent bonds between carbon-chain polymers and FCPI macromolecules, as well as the reaction mechanism, have been proven using differential scanning photocalorimetry, ESR, FTIR and NMR spectroscopy [51,53,54,55,56].

The results of the thermomechanical analysis of the samples did not allow the determination of the glass transition temperatures for most of the polymers; therefore, the values of the glass transition temperatures are not given in Table 2 (TMA plots are presented in Supplementary Information, Figure S1). The effect of increasing the thermal oxidative stability by the addition of PI has already been observed for (co)polymers based on methyl methacrylate, ethyl acrylate, styrene, N-vinyl-2-pyrrolidone, etc. The onset of the polymers obtained tends to increase by 10–30 °C when FCPI is added to the initial monomer (Table 2). This is also characteristic of polymers based on diacrylates, which do not degrade by the unzipping mechanism and have a higher thermal stability compared to poly(dimethacrylates). Due to the higher conversion of diacrylates, their polymer compositions with FCPI do not show the first stage of mass loss in the range of 130–160 °C, which is observed for poly(dimethacrylates) with FCPI and corresponds to the evaporation of an unreacted monomer (Table 1, Supplementary Information, Figure S1).

The results obtained demonstrate the possibility of significantly improving the mechanical and thermal properties of poly[di(meth)acrylates] by dissolving FCPI in the initial monomer and subsequent polymerization in situ.

### 2.2. Formation and Thermal Stability of New Photo-Curable Coatings of Silica Optical Fiber

**Figure 3:**
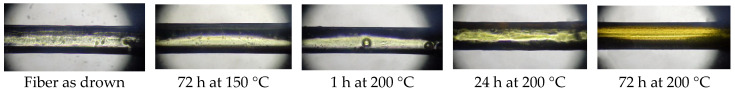
Photos of the optical fibers coated with poly(BDDA–FCPI_23%_) before and after thermal exposure.

**Figure 4:**
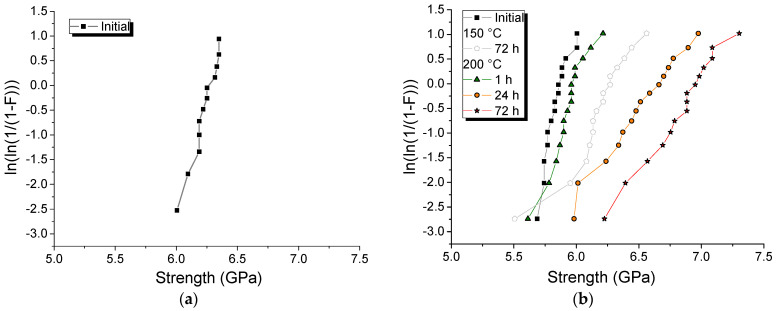
Strength of the optical fiber samples coated with poly(TTEGDA–FCPI_15%_) (**a**) and poly(BDDA–FCPI_23%_) (**b**) before and after thermal tests (the vertical axis represents the Weibull function; F is the cumulative fiber failure probability).

Taking into account the results of the study of the kinetics of the photopolymerization of di(meth)acrylates in the presence of dissolved PI, the viscosity of the solutions and the properties of some cured polymer networks, the compositions TTEGDA with 15 wt.% FCPI and BDAA with 23 wt.% FCPI were selected for the formation of primary protective coatings of silica optical fibers. The excellent adhesion of acrylate-based coatings to various substrates was an additional argument [22]. The solution of 15 wt.% FCPI in BDDA is characterized by a low viscosity (0.4 Pa·s), which leads to the formation of a defective coating on the fiber. Solutions of 15 wt.% FCPI in TTEGDA and 23 wt.% CPI in BDDA have a viscosity at 25 °C 5.2 and 4.8 Pa·s, respectively. These values are comparable to the viscosity of commercial DeSolite 3471-3-14 (9.3 Pa·s) and allow the formation of smooth homogeneous coatings (Figure 3). Primary protective coatings were formed on a special drawing tower (Supplementary Information, Figure S2) [59]. In order to assess the quality of the new coatings and their stability, the bending strength of the protected optical fibers before and after exposure to temperature was estimated using the well-known two-point bend test (Supplementary Information, Figure S3) [63]. The measured strength data of the optical fibers protected by poly(TTEGDA–FCPI_15%_) and poly(BDDA—FCPI_23%_) are presented as Weibull distributions in Figure 4.

As can be seen from the Figure 4, the initial strength of the silica optical fibers with new coatings varies in a small range from 5.7 to 6.0 MPa and from 6.0 to 6.7 MPa for poly(BDDA–FCPI_23%_) and poly(TTEGDA–FCPI_15%_), respectively. The slightly higher initial strength of the poly(TTEGDA–FCPI_15%_)-coated fiber can be explained by a higher polymerization rate of TTEGDA in the presence of 15 wt.% FCPI and, consequently, a lower content of unreacted monomer (Figure 1d,f). Given the high thermal stability of poly(TTEGDA–FCPI_15%_) (T_onset_ = 360 °C) and poly(BDDA–FCPI_23%_) (T_onset_ = 380 °C), it was of interest to evaluate the thermal stability of new silica optical fiber coatings prepared from them. Optical fibers with photo-cured coatings were exposed to different temperatures in a special thermal chamber.

Polyurethane acrylates form one of the most thermally stable UV-curable coatings, capable of operating up to 150 °C [64]. However, despite the high thermal stability of poly(TTEGDA–FCPI_15%_), its coating does not withstand annealing at 150 °C, which results in fiber adhesion and makes it impractical to test. At the same time, the exposure of a fiber with a coating of poly(BDDA–FCPI_23%_) to such a temperature, even for 3 days, only leads to an increase in fiber strength (Figure 4b). Apparently, when placing a fiber protected by poly(TTEGDA–FCPI_15%_) in the oven with a temperature of 150 °C, the coating softens due to the flexible ethoxy groups with unpolymerized acrylate fragments present in a weakly crosslinked polymer (curing time is about 1.5 s). This may be a reason for the fibers sticking together with such a coating. Most likely, the coating made of poly(BDDA–FCPI_23%_) is more stable due to the greater rigidity of the tetramethylene spacer, which leads to the preservation of its hardness.

The use of silicone rubber allows the working temperature to be increased to 200 °C [65]. As can be seen from the results presented in Figure 4b, the poly(BDDA–FCPI_23%_)-protected optical fiber withstands exposure to such a temperature. At the same time, the strength of the samples annealed at 200 °C for 1, 24 and 72 h increases only with increasing exposure time. The maximum average strength is observed after 72 h of heating and reaches 6.8 MPa, which is 17% higher than the initial value. However, the measured strengths vary over a wider range: from 6.2 to 7.3 MPa. Microscopic studies have revealed the appearance of bubbles in the coating after annealing at 200 °C for 1 h, which disappear after a longer exposure (Figure 3). A slight yellowing is observed after 24 h of exposure, which is more pronounced after 72 h treatment at 200 °C.

We believe that one of the reasons for the curing of the fibers is the further thermal polymerization of the BDDA. It is evident that after 1.5 s of photopolymerization, the coating contains some of the unreacted acrylate groups and a free BDDA in the polymer network forming a coating. The results obtained are comparable or superior to the previously described most highly thermostable UV-curable silica optical fiber coatings based on polyurethane acrylates, polysiloxane acrylates, etc. [26,66,67].

Based on the experimental results obtained, it can be concluded that high photopolymerization rates of compositions with the necessary viscosity, as well as satisfactory mechanical and thermal characteristics of the resulting polymers, are necessary but insufficient conditions for obtaining high-quality and effective coatings of silica waveguides. It is important to post-process the optical fibers after applying PPC and its rapid UV curing. The behavior of slightly crosslinked polymers based on TTEGDA and BDDA strongly depends on the nature of the spacer between the acrylate groups. This has a significant effect on the stability of the protective coating at temperatures of 150 °C and above.

## 3. Materials and Methods

### 3.1. Materials

Ethylene glycol dimethacrylate (EGDMA), triethylene glycol dimethacrylate (TIEGDMA), tetraethylene glycol dimethacrylate (TTEGDMA), 1,4-butanediol dimethacrylate (BDDMA), tetraethylene glycol diacrylate (TTEGDA) and 1,4-butanediol diacrylate (BDDA) were purchased from Sartomer Co. (Exton, PA, USA)and used without any purification. 4,4′-(Hexafluoroisopropylidene)diphthalic anhydride (6FDA) (98%), 4,4′-(hexafluoroisopropylidene)dianiline (6FpDA) (98%) and 4,4′-(9-fluorenylidene)dianiline (AFL) (98%) were purchased from Sigma-Aldrich (St. Louis, MO, USA) or TCI Chemicals (Zwijndrecht, Belgium) and additionally purified by vacuum sublimation. The photoinitiator 2,2-dimethoxy-2-phenylacetophenone (DMPA) (99%) (Sigma-Aldrich) was used as received. m-Cresol (99%) was distilled under reduced pressure. N-methyl-2-pyrrolidone and methanol were used without any purification.

### 3.2. Experimental

#### 3.2.1. Synthesis and Characterization of FCPI

The synthesis of FCPI was carried out according to the procedure described in detail in our previous works [57,59]. FCPI was prepared from 6FDA, AFL and 6FpDA (1.00:0.75:0.25 mol) by high-temperature one-step polycondensation in m-cresol. The inherent viscosity value was 0.36 dL/g (N-methyl-2-pyrrolidone, 25 °C). GPC results: Mn = 36 kDa, Mw = 56 kDa. Elemental analysis for C_166_H_80_F_30_N_8_O_16_: calculated: C 66.18%, H 2.68%, N 3.72%, F 18.92% and O 8.50%; found: C 65.83%, H 2.90%, N 3.75% and F 18.57%. FTIR: 1789 cm^−1^ (asym C=O of imide), 1727 cm^−1^ (sym C=O of imide), 1362 and 719 cm^−1^ (C-N of imide) and 1197–1106 cm^−1^ (C-F in CF3).

#### 3.2.2. Kinetics of Radical Photopolymerization

The kinetics of the radical photopolymerization of di(meth)acrylates in bulk and in solution with FCPI were studied by infrared spectrophotometry using a FT-801 Fourier-spectrometer (Simex, Novosibirsk, Russia) and an NPBO-A unit with a diamond element in the range of 400 to 1700 cm^–1^ with a resolution of 4 cm^−1^ using the method of broken total internal refraction. The wavelength of the excitation radiation was in the range of 365 to 405 nm; the radiation power was 150 mW/cm^2^. The PPCs were prepared by dissolving 5 wt.% (on the di(meth)acrylate) of the photoinitiator DMPA in the monomer or in the solution of the monomer with FCPI at 25 °C in air. The composition in a quantity of ~0.05 g was placed on the diamond element of the block and irradiated for three minutes in air. The conversion of di(meth)acrylate was calculated from the attenuation of the absorption bands of the C-H bond at the C=C bond based on at least three measurements. From the data obtained, the dependence of the monomer conversion on the irradiation of PPC was plotted and the values of the limiting conversion (P_lim_) were determined. The maximum photopolymerization rate (W_max_) for each PPC was determined from the tangent of the maximum slope angle to the kinetic curve.

#### 3.2.3. Polymer Films Preparation

Polymers for TGA, DMA and mechanical tests were prepared according to a previously described procedure [68] with minor modifications. The PPCs were prepared in the same way as for the study of photopolymerization kinetics. First, DMPA (5 wt.%) was dissolved in the corresponding di(meth)acrylate with stirring, and then FCPI. The prepared mixture was poured into a mold consisting of two flat silicate glasses with a Teflon spacer of 0.2 mm thickness and irradiation was started. The medium pressure mercury lamp DRT-240 (70 V, 240 W, LISMA SUE, Saransk, Russia) with a linear emission spectrum (fundamental wavelengths are 185, 254, 365, 405, 436, 546, and 578 nm) was used as a source of the ultraviolet irradiation. The UV energy density at the surface of the samples was 15 mW/cm^2^, determined by a radiometer combined Luxmeter + UV-radiometer TKA-PKM 06 (NTP TKA LLC, St. Petersburg, Russia). The sample was irradiated for 15 min and then placed in an oven at 80 °C for 30 min.

For example, 0.1105 g DMPA was first dissolved in 2.1 g EGDMA, and then 0.39 g FCPI was added to the solution while stirring. The resulting mixture was poured between two silicate glasses with a Teflon gasket 200 microns thick and irradiated for 15 min. The polymer between the glasses was additionally heated for 30 min at 80 °C, after which it was cooled and removed from the mold.

#### 3.2.4. Fiber Coatings Fabrication

The production of the fiber coatings was realized on a special drawing tower. The general scheme of the drawing is shown in Figure S2 (Supplementary Information). The fibers were drawn from a quartz preform using a standard SG Controls Ltd. (Cambridge, UK) system. The draw speed was 20 m/min, and the fiber diameter was ~120 microns. The photoactive composition was applied using an open die with a hole diameter of 280 microns through which the fiber was passed. Polymerization was carried out using an Optogear ultraviolet diode unit. The central wavelength of the diode laser was 393 nm, and the spectral width was 15 nm. The height of the UV block was 0.5 m, corresponding to an exposure time of 1.5 s. The diameter of the fiber before and after coating was measured with laser equipment. The average thickness of the coatings was 60 microns. The coated fiber was wound onto a capstan.

### 3.3. Characterization

The inherent viscosity (η_inh_) was measured for the solution of 0.05 g of FCPI in 10 mL N-methyl-2-pyrrolidone at 25.0 °C using an Ostwald capillary viscometer. The molecular weight characteristics of FCPI were analyzed on a 1200 Infinity gel permeation chromatograph (Agilent Technologies, Santa Clara, CA, USA) equipped with a PLgel 5 μm MIXED-D column (Agilent Technologies), PLgel 5 μm (Agilent Technologies) precolumn and an integrated refractive index detector. The system was operated at 50 °C and 1.0 mL/min flow using 0.1 M Li(CF_3_SO_2_)_2_N solution in DMF as an eluent. Poly(methyl methacrylate) standards (EasiVial PM, Agilent Technologies, Mp = 550–1558 × 10^3^) were used to perform calibration. The FTIR spectra of FCPI were recorded on a Bruker Vertex 70v FTIR-spectrometer (Billerica, MA, USA).

Rheological studies were conducted on an Anton Paar MCR 302 rheometer (Graz, Austria), in the constant shear rate (over the range of 1–500 s^−1^) plane–plane measuring mode, plane diameter 25 mm.

Thermomechanical analysis (TMA) was performed with a thermomechanical analyzer TMA/SDTA 2+ LN/600 (Mettler-Toledo, Greifensee, Switzerland) at a heating rate of 5 °C/min from 25 to 400 °C with constant load 1 N using a quartz stem with a ball point tip (diameter 3 mm). Thermogravimetric analysis (TGA) was performed by Thermoanalyzer Shimadzu DTG-60H (Kyoto, Japan) on samples with a weight of about 5 mg at a heating rate of 10 °C/min in air. The onset weight loss temperature (T_onset_) was defined from the intersection of two tangents on the TGA curve.

The tensile strength (σ), modulus of elasticity (E) and elongation at break (ε) were determined according to ISO 527:3:2018 [69]. Each polymer film was cut into 0.2 × 10 × 150 mm strips using a blade. Tensile tests were performed on an Autograph AGX-V 5 kN (Shimadzu, Tokyo, Japan) universal testing machine at a strain rate 5 mm/min. The modulus of elasticity was calculated from the slope of the linear area. The data presented were averaged over five samples for each composition.

A custom-made two-point bending setup was used [63] to measure the bending strength of the optical fiber samples with the faceplate rate of 0.1 mms^−1^. Polam L-213M optical microscope (LOMO JSC, St. Petersburg, Russia) was used to study fiber samples.

## 4. Conclusions

A new approach to the development of multicomponent curable compositions has been proposed. The excellent solubility of FCPI in different di(meth)acrylates allows the formulation of a variety of compositions, including photocurable ones. The viscosity of the initial composition can also be easily tuned by changing the monomer used and the FCPI content. The effect of FCPI on the photopolymerization rate and conversion of di(meth)acrylate, which depend on the type of monomer, spacer length and FCPI content, has been established. It was found that the introduction of FCPI (15 or 23 wt.%) into the initial monomers provides the resulting polymers with an increase in the tensile strength up to 2.2 times, modulus of elasticity up to 2 times, elongation at break up to 2.2 times and weight loss onset temperature up to 30 °C compared to unmodified carbon-chain homopolymers. The most suitable solutions of 15 wt.% FCPI in TTEGDA and 23 wt.% FCPI in BDDA have been tested in the manufacture of primary protective coatings for silica optical fibers. It is shown that the strength of a fiber coated with poly(BDDA–FCPI23%) only increases (from 5.9 MPa to 6.9 MPa) after prolonged annealing at 200 °C (72 h), indicating a significant thermal oxidative stability of this coating in air and its excellent adhesion. The developed approach provides the opportunity to adjust the properties of the initial photopolymerizing composition and the cured polymer, opening up wide possibilities in the production of functional materials for various applications.

## Figures and Tables

**Table 1:** Photopolymerization parameters of di(meth)acrylates and their compositions with FCPI (photoinitiator DMPA—5 wt.%).

Photopolymerizing Composition	W_max_ × 10^2^, s^−1^	P_lim_, %
EGDMA	2.38	64
EGDMA–FCPI_15%_	1.86	59
TIEGDMA	3.37	77
TIEGDMA–FCPI_15%_	2.93	70
TTEGDMA	4.95	98
TTEGDMA–FCPI_15%_	4.35	94
TTEGDA	6.81	100
TTEGDA–FCPI_15%_	8.46	100
BDDMA	2.34	75
BDDMA–FCPI_15%_	2.64	70
BDDA	7.51	98
BDDA–FCPI_15%_	7.66	97
BDDA–FCPI_23%_	3.55	86

**Table 2:** Mechanical and thermal properties of polymers.

Sample	σ, MPa	E, GPa	ε, %	T_onset_, °C
Poly(DeSolite 3471-3-14)	21.6	0.1	81.0	290
Poly(EGDMA)	20.9	2.0	1.1	260
Poly(EGDMA–FCPI_15%_)	Brittle			130, 290
Poly(TIEGDMA)	24.6	1.6	1.8	240
Poly(TIEGDMA–FCPI_15%_)	37.7	1.7	3.1	160, 240
Poly(TTEGDA)	19.4	0.5	17.5	330
Poly(TTEGDA–FCPI_15%_)	24.6	1.1	4.9	360
Poly(BDDMA)	24.5	2.1	1.4	260
Poly(BDDMA–FCPI_15%_)	Brittle			140, 270
Poly(BDDA)	20.2	1.5	1.6	370
Poly(BDDA–FCPI_23%_)	44.0	1.9	3.6	380

## Data Availability

Data is contained within the article (and Supplementary Materials).

## Supplementary Materials

The following supporting information can be downloaded at: https://www.mdpi.com/article/10.3390/ijms25105494/s1. References [70,71] are cited in the Supplementary Materials.
