# Supplementary material for: Soluble Fluorinated Cardo Copolyimide as an Effective Additive to Photopolymerizable Compositions Based on Di(meth)acrylates: Application for Highly Thermostable Primary Protective Coating of Silica Optical Fiber
Source: Int J Mol Sci. 2024 May 17;25(10):5494. doi: 10.3390/ijms25105494 (PMC11122490; doi:10.3390/ijms25105494)
Supplement: Supplementary file 1 [file ijms-25-05494-s001.zip › ijms-2992489-supplementary.pdf]

Supplementary Information to Article

Soluble fluorinated cardo copolyimide as an effective additive to photopolymerizable compositions based on di(meth)acrylates: application for highly thermostable primary protective coating of silica optical fiber

Dmitriy A. Sapozhnikov [1*], Olga A. Melnik [1], Alexander V. Chuchalov [1], Roman S. Kovylin [2], Sergey A. Chesnokov [2], Dmitriy A. Khanin [1], Galina G. Nikiforova, Alexey F. Kosolapov [3], Sergey L. Semjonov [3], Yakov S. Vygodskii [1]

[1] A. N. Nesmeyanov Institute of Organoelement Compounds, Russian Academy of Sciences, Vavilov str. 28, Moscow 119334, Russia
[2] G. A. Razuvaev Institute of Organometallic Chemistry, Russian Academy of Sciences, Tropinin str. 49, Nizhniy Novgorod 603950, Russia
[3] National Research Lobachevsky State University of Nizhniy Novgorod, Gagarin av. 23, Nizhniy Novgorod 603022, Russia
[4] Prokhorov General Physics Institute of the Russian Academy of Sciences, Dianov Fiber Optics Research Center, Vavilov str. 38, Moscow 119333, Russia
* Correspondence: ssddaa@ineos.ac.ru

Table of Contents

TMA and TGA results……………..………………………………………………………….…….…….…….…….……..S2

Fiber drawing scheme, two-point bend failure strain measurement and calculation……….…….…….…….……..…….S3

References…………………….……………..…………………….……………...……………...……………...……………...…...S4

TMA and TGA results

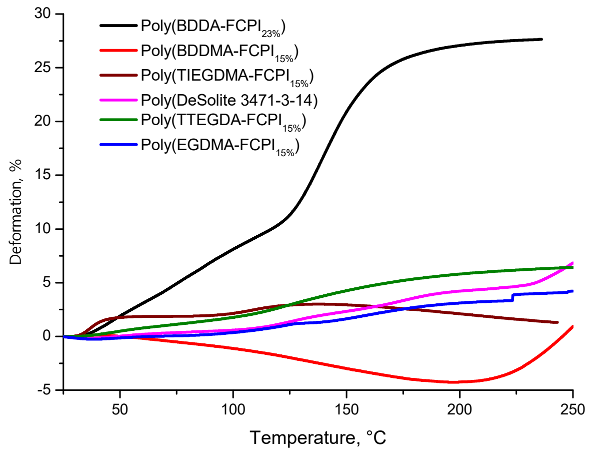

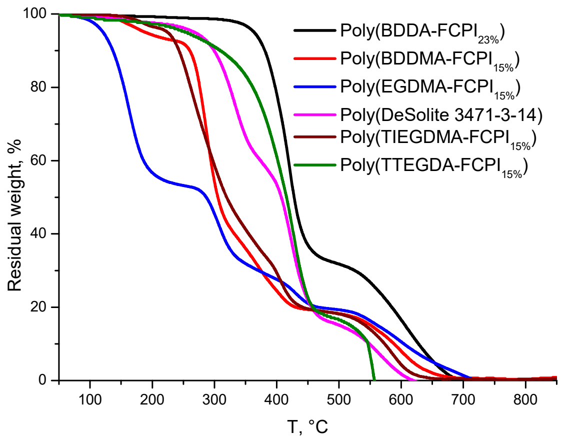

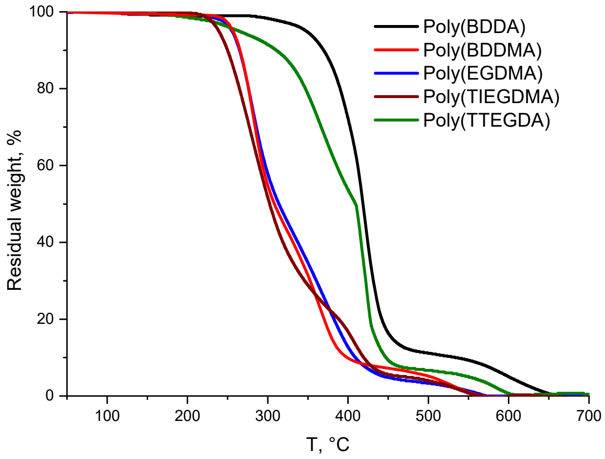

Figure S1. TMA and TGA results of polymers.

Fiber drawing scheme, two-point bend failure strain measurement and calculation

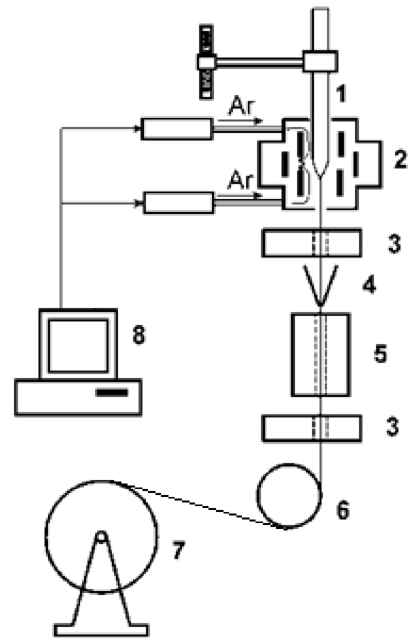

Figure S2. A general scheme of fiber drawing involving a conventional coating process (1 – silica preform, 2 – furnace, 3 – laser measuring system of diameter, 4 – die, 5 – UV block, 6 – pulling roller, 7 – reel, 8 – control system).

In a two-point bending test (Figure S3) [1], a section of glass fiber, diameter d (μm), is bent into a U-shape between two parallel faceplates, one of which travels towards the second at a constant faceplate velocity (v_{fp}), compressing the 'U' until failure. The distance between the axes of non-curved sections of the fibers at failure (Δ, μm) is recorded, and the maximum elongation (ε_{max}) is then calculated from:

$$\varepsilon_{max} = 1.219 * \frac{d}{\Delta_1} - 1.137 * \left(\frac{d}{\Delta_1}\right)^2$$

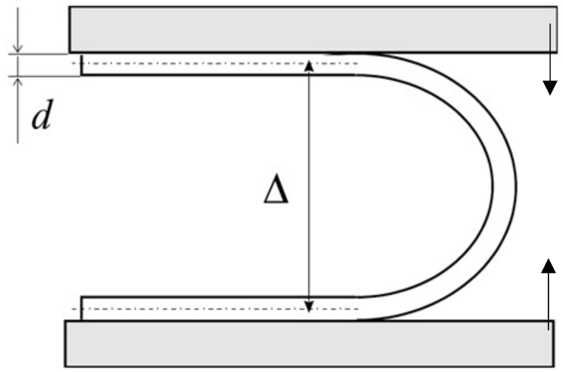

Figure S3. Schematic diagram of a two-point bend test.

Thus, it is possible to calculate the failure strain:

$\sigma_{max} = \varepsilon_{max} E$,

where E is the Young's modulus of quartz glass. Given that the Young's modulus depends on the relative elongation, we obtain the final expression for failure strain:

$\sigma = \varepsilon E_0 (1 + \frac{\propto \varepsilon}{2})$,

where E_0 = 74 GPa, α = 6.9.

Experimental results are presented in the form of Weibull plots [2].

References

1. Z. Tang, Two-point bend studies of glass fibers, Missouri University of Science and Technology, 2011. https://core.ac.uk/download/pdf/229270761.pdf

2. NIST/SEMATECH e-Handbook of Statistical Methods. https://www.itl.nist.gov/div898/handbook/eda/section3/eda33u.htm
